# Clinical outcomes of implantation of posterior chamber phakic intraocular lens for pathologic and non-pathologic myopia

**DOI:** 10.1186/s12886-023-02890-9

**Published:** 2023-04-21

**Authors:** Lin Caixia, Bai Yawen, Fang Yuxin, Li Xiaoxia, Wang Yuhan, Yang Ke, Qiao Liya

**Affiliations:** 1 Beijing Tongren Eye Center, Beijing Tongren Hospital, Capital Medical University, No. 1 Dong Jiao Min Xiang Street, Dongcheng District, Beijing, 100730 People’s Republic of China; 2 Ophthalmology Department, Beijing Shijitan Hospital, Capital Medical University, No. 10 Tieyi Road, Yangfangdian, Haidian District, Beijing, 100038 People’s Republic of China

**Keywords:** Posterior chamber phakic intraocular lens, Pathologic myopia, Non-pathologic myopia

## Abstract

**Background:**

To compare the clinical outcomes of posterior chamber phakic intraocular lens (pIOL) implantation for non-pathological myopia and pathological myopia.

**Methods:**

This retrospective case series study which were conducted in Beijing Tongren Eye Center between July 2017 and Oct 2021 comprised 192 eyes of 100 consecutive patients undergoing pIOL implantation. Eyes were divided into two groups based on having pathological myopia or not. Predictability, efficacy, safety, and adverse events were compared at 6 months after pIOL implantation.

**Results:**

Our study included 86 non-pathological myopes (171 eyes, group1) and 14 pathological myopes (21eyes, group2) to analysis. The average ages were 25.5 and 33.0, respectively, and the spherical equivalent (SE) were -9.31D and -17.50D pre-operation. Six months after pIOL implantation, the SE were 0.00 and -0.50, respectively, and the refraction changes were statistically significant (*P* ≤ 0.05). Six months after surgery, 76.92% and 80.41% were within ± 0.50 D of the target and 92.31% and 95.88% were within ± 1.00 D. All eyes had unchanged BCVA or gained 1 or more lines in both groups and mean BCVA both improved a line 6m after operation. The efficacy index in the two groups were 0.95 and 0.88 and the safety index were 1.20, 1.33, respectively which was significantly different (*P* ≤ 0.05). Over the 6-month follow-up, no cataract, pigment dispersion glaucoma, pupillary block, or other vision-threatening complications happened, either.

**Conclusions:**

The pIOL performed well for the correction of both non-pathological and pathological myopia throughout the 6-month observation period. The clinical outcomes of pIOL implantation for non-pathological myopia are essentially equivalent to those for pathological myopia.

## Background

Except for spectacles and contact lenses, there are many surgical options to correct myopia [1, 2]. Clear lens extraction (CLE), one procedure, has been used to treat high myopia for a long time [3, 4], however, after extracting the clear lens, people would lose natural accommodation. Refractive surgery, including laser-assisted in-situ keratomileusis (LASIK), small incision lenticule extraction and so on [5–7] has been widely accepted as a safe and effective surgical method for myopic correction, however, patients with high myopia or thin corneas have the risk of developing halos, glare and keratoectasia after undergoing refractive surgery. Besides, a large amount of laser ablation may lead to the decrease of superior intrinsic corneal optical performance.

In recent two decades, phakic intraocular lens (pIOL) implantation has gained widespread popularity as an effective refractive option for surgical correction of moderate to high ametropia [8–10]. Especially, the model V4c Visian Implantable Collamer Lens which was designed with a central hole of 0.36 mm could correct moderate or high myopia with little complications such as anterior subcapsular cataract, increased intraocular pressure (IOP), endothelial cell loss, pigment dispersion, pupillary block, and glaucoma. Besides, pIOL is a reversible procedure that could improve visual acuity and have excellent refractive stability while preserving accommodation.

Up to now, many clinical researches [11–17] have proved pIOL is a safe, effective and predictable procedure to correct moderate or high myopia. Nevertheless, there are few studies reporting clinical outcome of pIOL for pathological myopia. The aim of our research was to demonstrate the predictability, efficacy and safety of the model V4c pIOL to correct non-pathological and pathological myopia and to analyze the possible occurrence of adverse events at 6m after operation.

## Patients and methods

A retrospective chart review of a consecutive clinical case series study performed at Eye Center, Beijing Tongren Hospital, China between July 2017 and Oct 2021, consisting of 86 non-pathological myopia patients (171 eyes) and 14 pathological myopia (21 eyes) with Visian Implantable Collamer Lens (model V4c pIOL). This study followed the tenets of the Declaration of Helsinki and was approved by the institutional review board of Beijing Tongren Hospital. Written informed consent was obtained from all patients after they received a full explanation of the nature and possible consequences of the study.

The inclusion criteria were 1) age older than 18 years old, 2) a clear central cornea, 3) myopia less than -0.5D, 4) stable refraction for at least two years. The exclusion criteria included 1) anterior chamber depth (ACD) from the corneal endothelium of less than 2.8 mm, 2) endothelial cell density (ECD) less than 2000 cell/mm^2^, 3) mesopic pupil larger than 7.0 mm, 4) cataract, macular degeneration or retinopathy, retinal detachment, glaucoma, neuro-ophthalmic disease, amblyopia, and a history of ocular inflammation.

### Preoperative assessment

Before pIOL implantation, patients had a complete ophthalmologic examination, which included uncorrected distance visual acuity (UDVA), best corrected visual acuity (BCVA), manifest refractions, noncontact intraocular pressure (Canon), slit lamp examination, corneal topography (Oculus pentacam HR), pachymetry (Lenstar LS 900), ECD measurement (Specular microscope, SP.3000P), fundus photographs (Canon CR-2 AF, Japan), and binocular indirect ophthalmoscopy through dilated pupils.

### Phakic intraocular lens

The V4c Visian Implantable Collamer Lens model used in this study is a new model that has an artificial hole of 0.36 mm diameter in the optic center which is designed to improve the circulation of aqueous humor and eliminate the need for preoperative laser iridotomy or intra-operative peripheral iridectomy. Details about the model V4c pIOL have been described previously [11]. In all eyes, emmetropia or the minimum myopia dioptor was selected as the postoperative target refraction. The pIOL power calculation was performed using the modified vertex formula of the pIOL power table software which was provided by the manufacturer. The pIOL size was individually chosen according to the horizontal white-to-white distance and ACD and also following the pIOL manufacturer’s recommendations. The pIOL was implanted by the same experienced doctor (Q.L.Y) uneventfully according to the pIOL implantation technique [12].

### Fundus photograph classification

**Fig. 1:**
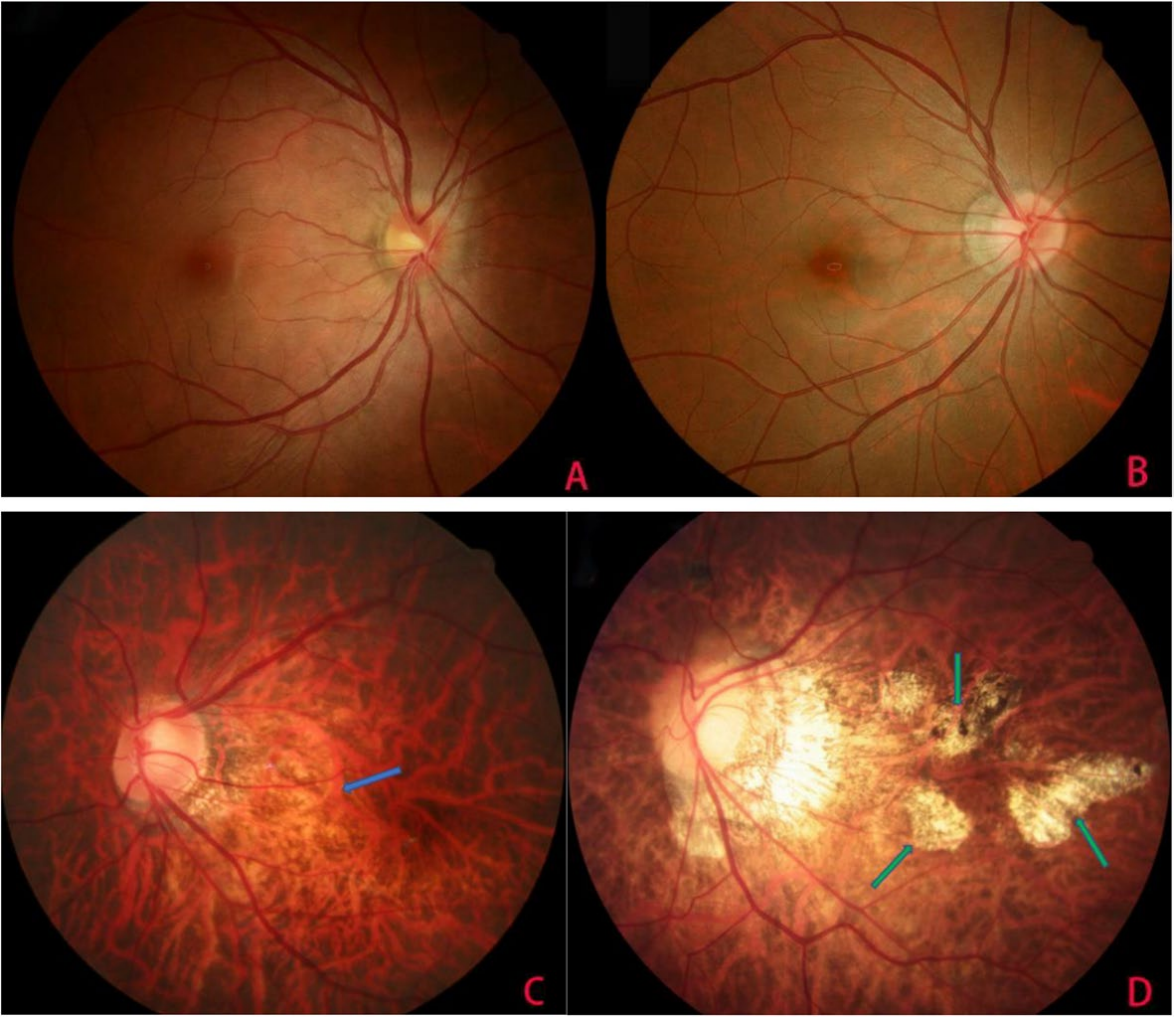
**A** Normal fundus: a female, 27-year-old, right eye, -7.25D. **B** Tessellation (well-defined choroidal vessels can be observed clearly around the fovea as well as around the arcade vessels), a female, 31-year-old, right eye, -7.75D. **C** Diffuse chorioretinal atrophy (the posterior pole of an eye with diffuse chorioretinal atrophy appears yellowish-white, the area pointed by blue arrow), a male, 33-year-old, left eye, -21.0D. **D** Patchy chorioretinal atrophy (patchy chorioretinal atrophy appears as well defined, grayish-white lesions, the area pointed by green arrows), a male, 29-year-old, left eye, -14.75D

According to fundus photographs we put the patients into non-pathological myopia (group 1) and pathological myopia (group 2), those with normal fundus or tessellation (Fig. 1A and B) were classified into group 1, those with diffuse chorioretinal atrophy (Fig. 1C), patchy chorioretinal atrophy (Fig. 1D), macular atrophy, lacquer cracks or myopic choroidal neovascularization (CNV) at the posterior pole were classified into group 2. Because Fuchs spot is a pigmented spot representing the scarring phase of myopic CNV, thus we put Fuchs spot into the myopic CNV category [18]. Fundus photographs were graded by two graders (L.C.X., B.Y.W) who had been trained for pathological myopia grading carefully. And the agreement in grading pathological myopia between them was excellent (weighted kappa values for all myopic maculopathy features were ≥ 0.8). When grading fundus photographs, if there was disagreement between the two graders, a third grader (F.Y.X) who had a lot of experience in grading pathological myopia also graded the photograph. If there was still no agreement after discussion, a retinal specialist (Q.L.Y) reassessed the relevant photographs and made a final diagnosis.

### Statistics

Statistical analyses were performed using SAS9.3. Patients with at least one eye satisfying the inclusion and exclusion criteria were enrolled in the study. All the statistical analyses were performed based on eyes. For the continuous outcomes, median and quartile ranges were presented due to non-normal distributions. For the categorical outcomes, the frequency and percentage were reported. To adjust the relationship between eyes, generalized estimated equation was used for all statistical comparisons. Patients were considered as the cluster, which had two levels (eyes). The correlation between measurements for the same subject was setting as exchangeable. Considering confounding effects, all the comparisons were further adjusted age and gender. Visual acuity data were converted to logMar values. Differences were considered statistically significant when the *P* value was less than 0.05.

## Results

Our study included 86 non-pathological myopes (171 eyes, group1) and 14 pathological myopes (21eyes, group2) to analysis. All the pathological myopes had diffuse and/or patchy chorioretinal atrophy, and didn’t have macular atrophy or other pathological changes. Table 1 showed demographics and pIOL characteristics (pre-operation) of non-pathological and pathological myopia patients. The average ages of these two groups were 25.5 (22, 30) and 33 (29, 43), respectively, and patients in group 1 were much younger than those in group 2 and the difference was significant (*P* ≤ 0.05). There were less males in group 1 than in group 2 (18.5% (10) vs 54.6% (6)). UCVA in group 1(3.22 (2.59, 3.91)) was poorer than that in group 2 (2.30 (2.30, 3.51)), however the difference was not significant. While BCVA in group 1(0.00(-0.18, 0.00)) was better than that in group 2(0.51 (0.11, 0.69)), and the difference was significant. The spheric, cylinder and SE in group 1 were much higher than those in group 2(-8.63D, -1.25D, -9.31D vs -17.5D, -1.5D, -17.5D), and there was a significant difference. The SE pIOL residual in group 1 was lower than that in group 2(-0.04D vs -0.15D, *P* = 0.0491), however the difference was not significant after adjusted age and gender (*P* = 0.1642). While there was no significant difference in VA, IOP, RF, RS, RM, white to white and ECD between the two groups.

**Table 1:** Demographics and ICL characteristics (pre-operation) of patients in group1 and 2

Characteristic	Group1 (*N* = 171 eyes)	Group2 (*N* = 21 eyes)	*P* value	*P* *
Age	25.5 (22, 30)	33 (29, 43)	0.0182	
Male	10 (18.5%)	6 (54.6%)	0.0200	
UCVA (logMAR)	3.22 (2.59, 3.91)	2.30 (2.30, 3.51)	0.5092	0.2109
BCVA (logMAR)	0.00 (-0.18, 0.00)	0.51 (0.11, 0.69)	< 0.001	< 0.001
IOP	15.50 (13.75, 17.30)	18.00 (14.50, 19.00)	0.2272	0.3936
Spheric	-8.63 (-10.75, -6.75)	-17.50 (-18.50, -13.50)	< 0.001	< 0.001
Cylinder	-1.25 (-1.75, -0.75)	-1.50 (-3.00, -1.50)	0.0343	0.0318
SE	-9.31 (-11.75, -7.25)	-17.50 (-18.75, -14.75)	< 0.001	< 0.001
SE pIOL residual	-0.04 (-0.14, 0.04)	-0.15 (-0.52, -0.08)	0.0491	0.1642
RF	7.77 (7.63, 8.00)	7.84 (7.74, 7.97)	0.5071	0.4815
RS	7.52 (7.38, 7.70)	7.57 (7.47, 7.84)	0.9634	0.8423
RM	7.63 (7.51, 7.83)	7.66 (7.63, 8.11)	0.2924	0.2897
White to White	11.86 (11.60, 12.09)	11.85 (11.66, 11.93)	0.5373	0.7090
ECD	2871.5 (2731.0, 3060.0)	3050.5 (2687.5, 3148.5)	0.6436	0.3900

The parameters are presented as mean (lower quartile, upper quartile) and number (percent), *UCVA* stands for uncorrected visual acuity, *BCVA* stands for best corrected visual acuity, *IOP* stands for intraocular pressure, *SE* stands for spherical equivalent, *RF* stands for corneal flat axil radius of curvature, *RS* stands for corneal steep axil radius of curvature, *RM* stands for mean corneal radius of curvature, *ECD* stands for endothelial cell density. *P* * stands for *P* value after adjusting for age and gender

Ocular parameters in the two groups 6 months (6m) after pIOL implantation were shown in Table 2, and from the table we can see that 6m after operation, patients with non-pathological myopia had better UCVA and BCVA (0.00, -0.18) than those with pathological myopia (0.51, 0.00), and the differences were significant. The spheric lens between the two groups (-0.13D vs -0.50D) was significantly different, however, after adjusted for age and gender, the difference was not meaningful further. The SE was also significantly different between the two groups (0.00D vs -0.50D). And there was no significant difference in IOP, cylinder, RF, RS, RM, white to white and ECD between the two groups.

**Table 2:** pIOL characteristics (post operation) of pathological and non-pathological myopia patients

Characteristic	Group1 (*N* = 171 eyes)	Group2 (*N* = 21 eyes)	*P* value	*P* *
UCVA (logMAR)	0.00 (-0.18, 0.11)	0.51 (0.00, 0.92)	< 0.001	0.0014
BCVA (logMAR)	-0.18 (-0.18, 0.00)	0.00 (0.00, 0.22)	0.0052	0.0114
IOP	15.45 (14.00, 17.40)	15.80 (14.30, 18.20)	0.2122	0.1244
Cylinder	-0.50 (-0.50, 0.50)	-1.00 (-1.00, -0.50)	0.1576	0.1576
Spheric	-0.13 (-0.25, 0.00)	-0.50 (-1.63, -0.13)	0.0451	0.1200
SE	0.00 (-0.38, 0.00)	-0.50 (-1.00, -0.50)	0.0133	0.0298
RF	7.77 (7.66, 8.14)	7.79 (7.68, 8.24)	0.4550	*0.3214*
RS	7.55 (7.45, 7.92)	7.64 (7.39, 7.94)	0.7242	0.5763
RM	7.70 (7.56, 8.02)	7.71 (7.51, 8.09)	0.5662	0.4215
White to White	11.86 (11.74, 12.20)	11.85 (11.68, 11.96)	0.4237	0.4236
ECD	2903.0 (2612.0, 2996.0)	2860.5 (2615.0, 3075.0)	0.5988	0.9598

The parameters are presented as mean (lower quartile, upper quartile) and number (percent), *UCVA* stands for uncorrected visual acuity, *BCVA* stands for best corrected visual acuity, *IOP* stands for intraocular pressure, *SE* stands for spherical equivalent, *RF* stands for corneal flat axil radius of curvature, *RS* stands for corneal steep axil radius of curvature, *RM* stands for mean corneal radius of curvature, *ECD* stands for endothelial cell density. *P* * stands for *P* value after adjusting for age and gender

Table 3 demonstrated ocular parameters pre-operation and 6m post operation in group 1. From the table, we can see that compared to pre-operation, patients had better UCVA and BCVA (3.22 and 0.00 vs 0.00 and -0.18) 6m after pIOL implantation, and the improvement was statistically significant. After pIOL plantation, spheric, cylinder and SE all obviously increased (from -8.63, -1.25D, -9.31D to -0.13D, -0.50D, 0.0D, respectively). In addition, RS became lager after operation compared to pre-operation (7.52 vs 7.55) and the difference was significant. While in IOP, RF, RM, white to white and ECD, there were no differences between pre-operation and post operation. From the table we also could see that ECD become lager post operation than pre-operation (2871.5vs 2903.0).

**Table 3:** Comparisons of ocular characteristics between pre-operation and 6m Post operation in group1 (*N* = 171 eyes)

Characteristics	pre-operation	Post operation	*P* value	P*
UCVA (logMAR)	3.22 (2.59, 3.91)	0.00 (-0.18, 0.11)	< 0.001	< 0.001
BCVA (logMAR)	0.00 (-0.18, 0.00)	-0.18 (-0.18, 0.00)	< 0.001	< 0.001
IOP	15.50 (13.75, 17.30)	15.45 (14.00, 17.40)	0.9335	0.9586
Spheric	-8.63 (-10.75, -6.75)	-0.13 (-0.25, 0.00)	< 0.001	< 0.001
Cylinder	-1.25 (-1.75, -0.75)	-0.50 (-0.50, 0.50)	< 0.001	< 0.001
SE	-9.31 (-11.75, -7.25)	0.00 (-0.38, 0.00)	< 0.001	< 0.001
RF	7.77 (7.63, 8.00)	7.77 (7.66, 8.14)	0.3798	0.3144
RS	7.52 (7.38, 7.70)	7.55 (7.45, 7.92)	0.0069	0.0061
RM	7.63 (7.51, 7.83)	7.70 (7.56, 8.02)	0.4062	0.4167
White to White	11.86 (11.60, 12.09)	11.86 (11.74, 12.20)	0.2425	0.2425
ECD	2871.5 (2731.0, 3060.0)	2903.0 (2612.0, 2996.0)	0.2520	0.2598

The parameters are presented as mean (lower quartile, upper quartile) and number (percent), *UCVA* stands for uncorrected visual acuity, *BCVA* stands for best corrected visual acuity, *IOP* stands for intraocular pressure, *SE* stands for spherical equivalent, *RF* stands for corneal flat axil radius of curvature, *RS* stands for corneal steep axil radius of curvature, *RM* stands for mean corneal radius of curvature, *ECD* stands for endothelial cell density. *P* * stands for *P* value after adjusting for age and gender

Table 4 displayed that compared to ocular parameters pre-operation, patients in group 2 had better UCVA and BCVA (2.30, 0.51 vs 0.51, 0.00), lower spheric and SE (-17.5D, -17.5D vs -0.5D, -0.5D) 6m after operation. Besides, after adjusted for age and gender, cylinder difference became significant between pre-operation and post operation (-1.5D, -1.0D). And there were no differences between pre-operation and post operation in IOP, RF, RS, RM, white to white and ECD.

**Table 4:** Comparisons of characteristics between pre-operation and post operation in group2 (*N* = 21 eyes)

Outcome	Pre-operation	Post operation	*P* value	P*
UCVA (logMAR)	2.30 (2.30, 3.51)	0.51 (0.00, 0.92)	< 0.001	< 0.001
BCVA (logMAR)	0.51 (0.11, 0.69)	0.00 (0.00, 0.22)	< 0.001	< 0.001
IOP	18.00 (14.50, 19.00)	15.80 (14.30, 18.20)	0.4960	0.6716
Spheric	-17.50 (-18.50, -13.50)	-0.50 (-1.63, -0.13)	< 0.001	< 0.001
Cylinder	-1.50 (-3.00, -1.50)	-1.00 (-1.00, -0.50)	0.2989	0.0244
SE	-17.50 (-18.75, -14.75)	-0.50 (-1.00, -0.50)	< 0.001	< 0.001
RF	7.84 (7.74, 7.97)	7.79 (7.68, 8.24)	0.7183	0.7436
RS	7.57 (7.47, 7.84)	7.64 (7.39, 7.94)	0.7989	0.7995
RM	7.66 (7.63, 8.11)	7.71 (7.51, 8.09)	0.3184	0.3213
White to White	11.85 (11.66, 11.93)	11.85 (11.68, 11.96)	0.8072	0.6368
ECD	3050.5 (2687.5, 3148.5)	2860.5 (2615.0, 3075.0)	0.2989	0.2988

The parameters are presented as mean (lower quartile, upper quartile) and number (percent), *UCVA* stands for uncorrected visual acuity, *BCVA* stands for best corrected visual acuity, *IOP* stands for intraocular pressure, *SE* stands for spherical equivalent, *RF* stands for corneal flat axil radius of curvature, *RS* stands for corneal steep axil radius of curvature, *RM* stands for mean corneal radius of curvature, *ECD* stands for endothelial cell density. *P* *stands for *P* value adjusted for age and gender

**Fig. 2:**
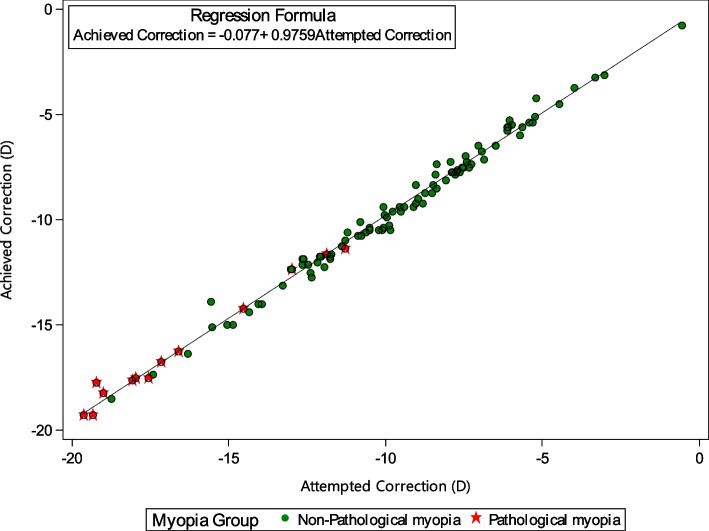
Predictability of mean SE (attempted versus achieved correction) 6 months after pIOL implantation. The continuous line represents the best linear fit to the data

Figure 2 showed a scatterplot of the attempted versus the achieved SE correction. Six months after surgery, 76.92% (10/13eyes) in group 1 and 80.41% (78 /97eyes) in group 2 were within ± 0.50 D of the target and 92.31% (12/13 eyes) and 95.88% (93/97 eyes) were within ± 1.00 D, respectively.

Clinical outcomes 6m after pIOL implantation in group 1 and group 2 were shown in Table 5. The efficacy index in the two groups were 0.95(0.83, 1.00) and 0.88(0.67, 1.00) and the safety index were 1.20(1.00, 1.20), 1.33(1.11, 1.43), respectively and the safety index between the two groups were significantly different. BCVA in the two groups both improved one line and the exact increase numbers were -0.18(-0.18, 0.00) and -0.29(-0.36, -0.11), separately. ECD increased in group 1 (0.01(-0.04, 0.04) while ECD loss happened in group 2 (-0.01(-0.02,0.07), however, the difference was not significant. The Arch in the two groups were 500.50 (360.50, 750.50) and 573.00 (333.00, 623.00), separately, and the difference was large, however, there was no clinical significance. There was more percentile (72.5% (71) vs 30.8% (4)) to have good VA (≥ 1.0) in group 1 than in group 2 and the difference were significant.

**Table 5:** Comparison of outcomes 6m after pIOL implantation in group1 and group2

Characteristic	Group1(*N* = 171 eyes)	Group2 (*N* = 21 eyes)	*P* value	P*
Efficacy	0.95 (0.83, 1.00)	0.88 (0.67, 1.00)	0.1275	0.3972
Safety	1.20 (1.00, 1.20)	1.33 (1.11, 1.43)	0.0466	0.0413
BCVA Increase (logMAR)	-0.18 (-0.18, 0.00)	-0.29 (-0.36, -0.11)	0.0592	0.0680
BCVA Increase (Line)	1.00 (0.00, 1.00)	1.00 (0.00, 1.00)	0.1654	0.1916
ECD Loss	0.01 (-0.04, 0.04)	-0.01 (-0.02, 0.07)	0.8832	0.0728
Arch	500.50 (360.50, 750.50)	573.00 (333.00, 623.00)	0.3894	0.1690
VA (> = 1.0)	71 (72.5%)	4 (30.8%)	0.0059	0.0292

The parameters are presented as mean (lower quartile, upper quartile) and number (percent), BCVA stands for best corrected visual acuity, *ECD* stands for endothelial cell density, *VA* stands for visual acuity. *P* *stands for *P* value adjusted for age and gender

**Fig. 3:**
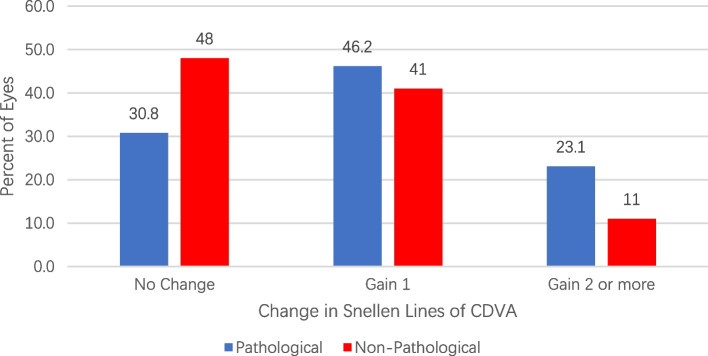
Changes in corrected distance visual acuity (CDVA) 6 months after pIOL implantation in eyes with non-pathological myopia and pathological myopia

Figure 3 showed the changes in BCVA in group 1 and group 2 six months after pIOL implantation. From the figure we could see that no eye lost 1 or more lines, 30.8% and 48% did not change from preoperatively, 46.2% and 41% gained 1 line, 23.1% and 11% gained 2 lines or more, respectively, in the two groups. There was more percentile in group 1 to gain 1 line or more compared to that in group 2, yet the difference was not significant.

There were no intraoperative complications, and no eye required pIOL explantation or repositioning in the two groups. Over the 6-month follow-up, no cataract, pigment dispersion glaucoma, pupillary block, or other vision-threatening complications happened, either.

## Discussion

The V4c Visian Implantable Collamer Lens (pIOL) has been improved to have good predictability, stability, efficacy, and safety for the correction of moderate to high myopia in previous studies [12, 15, 19]. However, there is few researches previously to study how the pIOL implantation to correct pathological myopia. The present study showed that the pIOL implantation is an effective treatment for both non-pathological and pathological myopia, with excellent safety and predictability throughout a 6-month observation period.

Our study demonstrated that spheric, cylinder and SE all increased significantly in both groups 6m after pIOL implantation and patients all get good UCVA (from 3.22 to 0.00 and from 2.30 to 0.51) and BCVA (from 0.00 to -0.18 and from 0.51 to 0.00). The UCVA and BCVA improvement may be contributed to that compared to frame glasses, pIOL has less spherical aberration, astigmatism and other off-axis aberrations, less prism effect, wider field of vision and smaller reduction of retinal imaging, thus after pIOL implantation patients can gain better VA no matter UCVA or BCVA. Compared with patients in group 1, those in group 2 had poorer UCVA and BCVA after pIOL implantation, the reason may be that patients with pathological myopia had pathological changes such as diffuse chorioretinal dystrophy which could decrease the resolution of retina, yet the difference is not significant.

In group 1, we found that RS became lager 6m after operation, this may be caused by during pIOL implantation, we need to make a transparent limbal incision which flattens the steep axis of the cornea and increases the radius of corneal curvature. Thus, we can design the surgical incision location combined with the corneal astigmatism axis to reduce corneal astigmatism during the operation.

Both the postoperative UDVA and BCVA in group 1 were significantly better than in group 2, possibly because patients in group 1 had lower rates of myopia than those in group 2. And there was more percentile who gained 1.0 or better UCVA in group 1 than in group 2, this may be due to patients with pathological myopia have some pathological change which affect the VA improvement after pIOL implantation.

We obtained stable (0.00 (-0.38, 0.00) and -0.50 (-1.00, -0.50)) and predictable refractive outcomes in both groups 6m after surgery, with 76.92% (10/13eyes) in group 1 and 80.41% (78 /97eyes) in group 2 within ± 0.50 D of the target and 92.31% (12/13 eyes) and 95.88% (93/97 eyes) within ± 1.00 D. The percentiles were similar to those of Kazutaka Kamiya’s research [20] and lower than those of other previous studies [11–13], the reason may be that we included some patients whose myopia were less than -18.0D, the lowest diopter of the pIOL.

The efficacy and safety of non-pathologic myopia and pathologic myopia were 0.95 and 0.88, 1.20 and 1.33, respectively, the efficacy in the two groups was comparable and the safety in group 2 was higher than that in group 1, which means patients with pathological myopia could get a lager BCVA improvement after pIOL implantation, the reason may be that patients with pathological myopia usually have higher myopia, therefore the aberration and reduction of retinal imaging were more obvious caused by frame glasses, after pIOL implantation, the visual improvement was more significant than those with non-pathological myopia. And the efficacy and safety of both non-pathologic myopia and pathologic myopia groups were similar to previous studies [11, 13, 15] which means that pIOL is an effective and safe operation to correct non-pathological myopia as well as pathological myopia. Previous studies [21–23] indicated that pIOL implantation provided long-term stability and good refractive outcomes for high myopia.

About ECD change, one common complication after pIOL implantation, we found that ECD decreased in group 2 while it increased in group 1 six months after pIOL implantation, however, the difference between pre-operation and post operation was little (1%) and not significant. the ECD increase may be caused by that the ECD in different corneal endothelial regions is different, and the measurement areas chosen before and after operation were different which led to the ECD increase after operation. In addition, the measurement error also played a certain role. And the specific reasons need to be further explored. About ECD reduction, Yan Ju et al. [24] found that the mean 3-month postoperative ECD decreased but had no statistically difference compared with the preoperative ECD after pIOL implantation. Whose result was similar to ours. Regarding the long-term endothelial cell loss, Jime´nez-Alfaro et al. [25] showed that the percentage of endothelial cells lost was 6.57% 2 years after surgery [26]. Pineda-Fernandez et al. [26] found 6.09% of endothelial cell loss 3 years after surgery. Kazutaka Kamiya et al. [17] reported that the percentage of endothelial cell loss was 3.7% 4 years postoperatively, and Alfonso et al. [21] stated that the total endothelial cell loss was 7.7% 5 years postoperatively. Akihito Igarashi et al. [22] found that the mean percentage of endothelial cell loss was 6.2% 8 years after surgery. Jae Hwan Choi et al. [16] found that the rate of ECD decrease was 4.8% at 10 years after surgery. However, no eyes reduced to less than 2000 cell/mm^2^ or had a significant loss over 30%. A decrease in the mean ECD in acute stage could be aggravated by endothelial damage during pIOL implantation, inflammation after implantation, or physical contact between the pIOL and corneal endothelium. And the main cause of endothelial cell loss over the long term is the aging process [16]. How the number of endothelial cells change over long time about our patients need to be observe.

In our research, we didn’t find any case with other postoperative complications including IOP rise (covering pupillary block) and cataract formation in both non-pathological and pathological myopia groups 6m after pIOL implantation. These findings were consistent with those of previous studies on pIOL implantation, even without laser or surgical peripheral iridotomy or iridectomy performed [15, 27, 28]. Kawamorita et al. [29] used computational fluid dynamics to demonstrate that hole pIOLs may improve the circulation of aqueous humor to the anterior surface of the crystalline lens which may prevent postoperative pupillary block and cataract formation. Previously Kamiya et al. reported that the incidence of cataract formation with V4 pIOL was 10.7% four years after pIOL implantation if traumatic cataract formation was excluded [17]. While, Packer et al. [30] in a Meta-analysis and review on Hole pIOL implantation including data on 1291 eyes followed for up to 5 years reported no incidence of asymptomatic anterior subcapsular cataract formation. And they also reported that the age at surgery and degree of myopia are risk factors for cataract formation after pIOL implantation. Jae Hwan Choi et al. [16] found that there was a higher cumulative incidence of lens opacity in patients with higher preoperative age or more severe preoperative myopia. And in the future more prolonged and careful follow-up researches are still required to determine the exact rate of cataract formation in Hole pIOL implantation eyes.

This study has several limitations. First, the study was performed in a retrospective way, a prospective randomised study would be ideal for confirming our results. Second, there were a relatively low patient population, especially in the pathological group, thus our data may have a possible selection bias. Third, a 6m of follow-up might not be sufficient to evaluate the long-term complications of a pIOL. The last but not the least, the number of patients in the pathological group was small and the pathological myopia mainly contained diffuse and patchy chorioretinal atrophy, how does other pathological myopia such as macular atrophy, lack cracks and so on with pIOL implantation, we don’t know. Nevertheless, to our knowledge, the current study was the first to evaluate refractive and visual outcomes of V4c pIOL for correction of non-pathological and pathological myopia.

In conclusion, our study supports the view that the pIOL performed equally well in correction of pathological myopia as it did in non-pathological myopia during the 6-month observation period. pIOL implantation for pathological myopia is clinically, if not statistically, equivalent to the results in non-pathological myopia in terms of the predictability, efficacy and safety. pIOL implantation is a viable surgical option for the treatment of pathological myopia.

## Data Availability

The datasets used and/or analysed during the current study are available from the corresponding author on reasonable request.

## Abbreviations

pIOL: Posterior chamber phakic intraocular lens
CLE: Clear lens extraction
LASIK: Laser-assisted in-situ keratomileusis
IOP: Intraocular pressure
ACD: Anterior chamber depth
ECD: Endothelial cell density
UDVA: Uncorrected distance visual acuity
BCVA: Best corrected visual acuity
CDVA: Corrected distance visual acuity
CNV: Choroidal neovascularization
SE: Spherical equivalent
